# Investigation of corneal hydration and the impact of cross-linking therapy on water retention using Brillouin spectroscopy, Raman spectroscopy and polarization-sensitive optical coherence tomography

**DOI:** 10.3389/fbioe.2025.1576809

**Published:** 2025-06-24

**Authors:** Jan Rix, Svea Steuer, Jonas Golde, Fadi Husein, Felix Lochmann, Steven Melcher, Gerald Steiner, Roberta Galli, Julia Walther, Frederik Raiskup, Ramin Khoramnia, Robert Herber

**Affiliations:** ^1^ TU Dresden, Faculty of Medicine Carl Gustav Carus, Medical Physics and Biomedical Engineering, Dresden, Germany; ^2^ TU Dresden, Faculty of Medicine Carl Gustav Carus, Department of Anesthesiology and Intensive Care Medicine, Clinical Sensoring and Monitoring, Dresden, Germany; ^3^ Fraunhofer Institute for Material and Beam Technology IWS, Dresden, Germany; ^4^ Department of Ophthalmology, Faculty of Medicine and University Hospital Carl Gustav Carus, TU Dresden, Dresden, Germany

**Keywords:** cornea, corneal cross-linking, Brillouin, Raman, PS-OCT, hydration

## Abstract

Recently, Brillouin spectroscopy has been proposed as a promising non-invasive tool to evaluate corneal biomechanics, e.g., during corneal cross-linking (CXL) treatment. However, the impact of corneal hydration on the Brillouin shift hampers straightforward interpretation of the measurements, especially when judging on the success of the CXL procedure. Therefore, in this work, we first quantify the effect of corneal (de)hydration on the Brillouin shift revealing that reliable measurements are only possible under constant hydration conditions, which was subsequently achieved by immersing porcine eyes in solution and waiting until an equilibrium state was reached. Investigations showed that Brillouin spectroscopy evaluates the CXL effect mainly indirectly via reduced water uptake, while polarization-sensitive optical coherence tomography evaluates the CXL effect directly via changes in collagen fiber alignment and is therefore insensitive to corneal hydration. Raman spectroscopy is not indicating any alterations in the molecular structure revealing that new cross-links are not created due to the CXL procedure. Compared to large water retention in balanced salt solution, the missing water uptake in dextran-based (16%) solution hampers the evaluation of the CXL effect by Brillouin spectroscopy.

## 1 Introduction

Keratoconus is an eye disease in which the cornea becomes thinner and cone-shaped (Rabinowitz, 1998). This unnatural corneal curvature results in astigmatism and a loss of visual acuity, which cannot be corrected by eyeglasses (Zadnik et al., 1996). While the underlying causes for this pathology are still subject of current research (Galvis et al., 2015; Karolak and Gajecka, 2017; Sugar and Macsai, 2012), corneal cross-linking (CXL) has been established as the standard treating method during the last 20 years, as it slows down or even stops corneal weakening (Raiskup et al., 2024; McGhee, Kim, and Wilson, 2015). In CXL, the corneas are soaked with the photosensitizer riboflavin and then irradiated with UV-A light to strengthen the collagen fibers and thus restore the original tensile strength of the cornea. Although CXL is routinely performed in clinics, the underlying molecular mechanisms are still subject of research (Hayes et al., 2013; McCall et al., 2010; O’Brart and David, 2014).

The most commonly used technique to evaluate the cross-linking effect is stress-strain extensiometry (Elsheikh and Anderson, 2005). Previous stress-strain investigations on porcine corneas have shown that CXL leads to a corneal stiffening by a factor of about 1.3 (Schumacher et al., 2011; Hammer et al., 2014). Consequently, corneal stiffening stabilizes the clinical progression of keratoconus in about 90% of cases (Koller et al., 2009; Caporossi et al., 2011). However, stress-strain measurements come along with an inherent shortcoming, as they are not performable *in vivo*. For this purpose, other techniques such as Scheimpflug-based air-puff tonometry (Vinciguerra et al., 2017) are currently being investigated whether they allow for *in vivo* assessment of CXL. Several *in vivo* studies have demonstrated a stiffening effect after CXL, particularly 1 month after treatment, using specific parameters of the device (Herber et al., 2023a; Herber et al., 2023b; Vinciguerra et al., 2019). In addition to these methods based on mechanical deformation, which might be unpleasant or painful for the patients and provide only general information about the cornea and not local changes, also full-optical Brillouin spectroscopy (BS) was proposed for the evaluation of CXL (Scarcelli et al., 2012; Scarcelli et al., 2013). Hereby, laser light is inelastically scattered by thermally excited sound waves in the tissue (Palombo and Fioretto, 2019). The occurring Brillouin frequency shift $\nu_{B}$ in the gigahertz range is related to the biomechanical properties via the speed of sound $v_{s}$ at which the sound wave propagates in tissue (Equation 1):

$$M^{\prime }=\rho v_{s}^{2}=\frac{\rho \;\lambda^{2}\;{\nu_{B}}^{2}}{4\;n^{2}\;\sin^{2}\left(\theta /2\right)}$$ (1)

where $M^{\prime }$ is the real part of the longitudinal modulus, ρ the mass density, λ the laser excitation wavelength, n the refractive index at the excitation wavelength and θ the scattering angle (Scarcelli et al., 2015). It should be emphasized that the Brillouin shift is related to the longitudinal modulus, whereas e.g., stress-strain measurements give access to the Young’s modulus. Although these both measures are not identical and describe slightly different measuring situations, they are correlated with each other (Seiler et al., 2018; Webb et al., 2017; Scarcelli et al., 2011).

In the past 15 years, BS was used in several studies addressing corneal biomechanics (Scarcelli et al., 2012; Scarcelli et al., 2013; Seiler et al., 2018; Webb et al., 2017; Scarcelli et al., 2011; Scarcelli et al., 2014; Shao et al., 2019; 2018; Lepert et al., 2016; Lopes and Ahmed, 2023; Eltony et al., 2022; Webb et al., 2020; Seiler et al., 2019). Key results are that BS is able to differentiate between normal and various pathological biomechanical properties as well as artificially induced alterations, e.g., due to CXL. During this wide and promising application of BS in cornea research, it was noticed that the Brillouin frequency shift is also affected by corneal hydration, which might hamper straightforward *ex vivo* measurements (Seiler et al., 2018; Shao et al., 2018; Antonacci et al., 2020). Wu et al. even questioned whether hydration instead of stiffness is measured in highly hydrated media (Wu et al., 2018). The hydration aspect is also known from other biomedical applications (Wu et al., 2019; Jannasch et al., 2021) or rather made use of e.g., by changing the osmotic conditions in order to regulate the water balance (Scarcelli et al., 2015; Zhang et al., 2023). For protecting the cornea from dehydration (especially after de-epithelization), a moist chamber was used in several *ex vivo* studies (Scarcelli et al., 2012; Scarcelli et al., 2014). However, it remains unclear on what time scale dehydration and rehydration processes take place. Thus, it is for example, questionable, whether the dehydration during sample preparation can be compensated by short-time rehydration. Since a non-equilibrium hydration comes along with Brillouin shift measurements, hydration should in any case be accounted for in order to investigate weak mechanical effects like CXL reliably.

Brillouin spectroscopy can be combined with Raman spectroscopy. Raman scattering is also excited using a laser. Variations in the polarizability of molecular bonds lead to a vibration of the atoms and the generation of Raman scattering. The Raman spectrum provides a high amount of information about the molecular structure and is therefore sometimes referred to as a molecular fingerprint.

A recent study by our group, in which Surface Enhanced Raman Spectroscopy (SERS) was used to examine the CXL, showed only minimal variations of the molecular structure (Melcher et al., 2023). The observed changes were assigned to side chains. However, they alone do not explain the mechanical stiffening of the cornea. In any case, SERS can only be used to examine areas on the surface that are only a few nanometers thick.

Optical coherence tomography (OCT) is a well-established imaging technique in ophthalmology e.g., to investigate age-related macular degeneration, retinal detachment and diabetic retinopathy (Al-Mujaini et al., 2013). Recently, it was shown that anterior segment OCT is able to detect the stromal demarcation line being an indicator for the effective CXL depth (Doors et al., 2009; Kymionis et al., 2016). However, the visibility of the demarcation line from the OCT intensity images is quite challenging and subjective. Using the tissue-induced alterations of polarized light as an additional contrast, it was recently demonstrated that polarization-sensitive OCT (PS-OCT) is able to reveal the CXL effect by analyzing the degree of polarization (DOP) (Ju and Tang, 2015). After CXL, the DOP is increased in the upper part of the cornea, wherefore it can be used for validation of the CXL effect, too. The authors explain the increased DOP by a denser and more compact packaging of the collagen fiber network. However, the connection to the underlying biochemical changes due to CXL treatment is still missing as well as the connection to mechanical stiffening and the hydration dependency of the DOP.

The aim of this study is threefold: i) to investigate the impact of corneal hydration on the Brillouin shift measurements ii) to find conditions at which the hydration is in an equilibrium state and iii) to evaluate the detectability of CXL by means of BS. RS and PS-OCT were used to better understand the molecular and microscopic mechanisms underlying CXL and to verify whether the CXL was successful. The overall aim of this study is to improve the understanding of CXL’s underlying molecular and microscopic mechanisms, which are responsible for corneal stiffening. This knowledge is on the one hand important to further optimize the CXL procedure, e.g., in terms of treatment time, UV light dose and treatment frequency. On the other hand, this study will support the development of future *in vivo* diagnostic approaches for CXL monitoring.

In a first part, this paper describes the time-dependence of corneal dehydration in order to show that reliable Brillouin shift measurements are only possible at constant hydration. This is in particular important for subsequent measurements of the CXL effect, which might otherwise be obscured. In a second part, the time-dependence of corneal hydration is studied in order to find the time after which an equilibrium hydration state is reached. This information is then used in the CXL measurements, where joint depth-dependent Brillouin spectroscopy, Raman spectroscopy and PS-OCT measurements are performed.

## 2 Materials and methods

### 2.1 Sample preparation

Porcine eyes were obtained from a local slaughterhouse. It should be emphasized that the animals were not killed for the purpose of this study, rather than the eyes were used as waste-products from meat industry. All eyes were measured within 12 h *postmortem*. For dehydration and hydration measurements, paired eyes, i.e., from the same animal, were used. After careful de-epithelization with a hockey knife, one eye was measured with BS, while the other one was examined with OCT. All measurements were carried out in an air-conditioned laboratory having about 50% relative humidity.

Paired eyes were also used for measurements of the CXL effect. After de-epithelization, one of the paired eyes was treated with a CXL protocol, i.e. 15 min riboflavin soaking followed by UV-A light (λ = 365 nm) irradiation. Here, the eyes were divided into two groups. Eyes of the first group were irradiated for 10 min with 9 mW/cm^2^ (5.4 J/cm^2^), whereas eyes of the second group were irradiated for 12 min with 15 mW/cm^2^ (10.8 J/cm^2^). The second of the paired eyes was only exposed to riboflavin, but not irradiated with UV-A light, and thus served as control sample. Instead of irradiation, it was rested for the same time. Afterwards, all eyes were placed for 15 min in balanced salt solution (BSS, Eye-lotion Balanced salt solution, Serumwerk Bernburg AG, Bernburg, Germany) to generate an equilibrium hydration state. All eyes were analyzed by combined Brillouin and Raman spectroscopy via a water-dipping objective and afterwards with PS-OCT. In a further experiment, paired eyes were placed in 16% Dextran solution (Dextran 500, Carl Roth GmbH, Karlsruhe, Germany) instead of BSS and treated with the 9 mW/cm^2^ protocol only. Since the 16% Dextran solution possesses a mass density close to or even a bit higher than that of the porcine eyes they tend to lift and swim in the solution hampering accurate measurements. Therefore, a metal ring was placed on the eyes as an additional weight in order to compensate for buoyancy while maintaining optical access to the cornea.

### 2.2 Combined Brillouin and Raman spectroscopy

**FIGURE 1:**
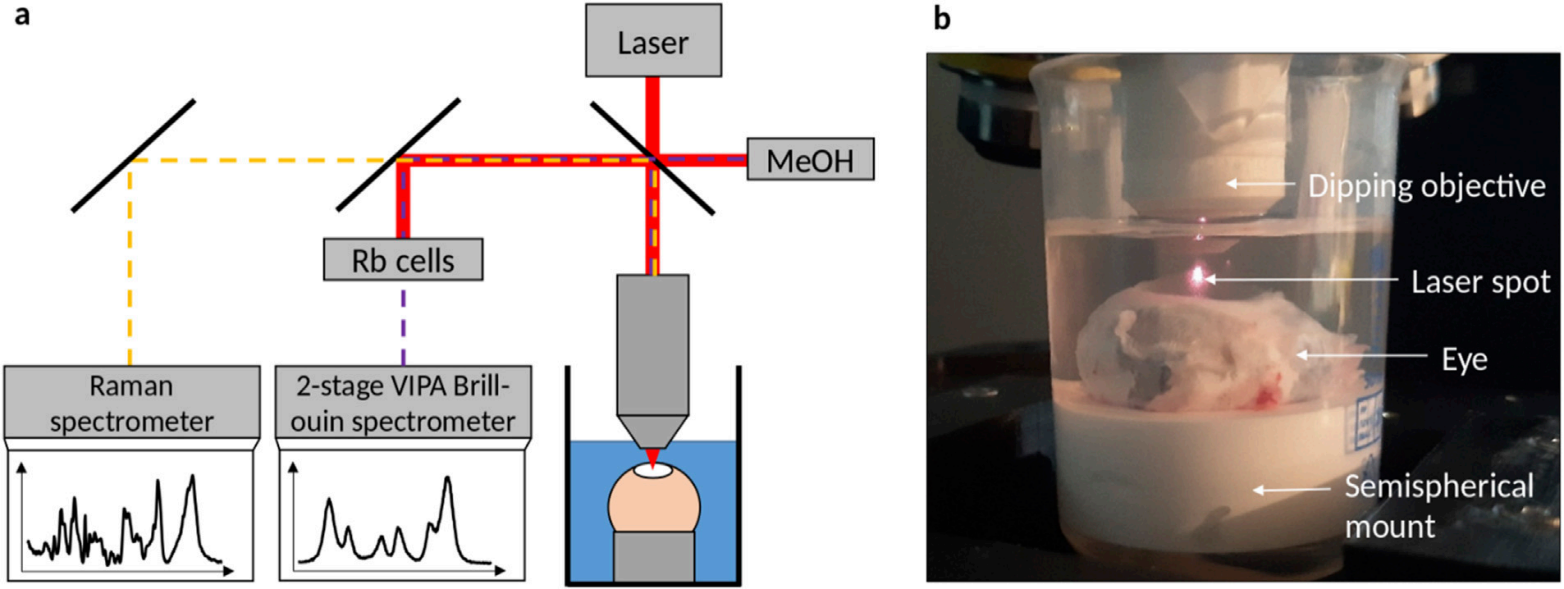
**(a)** Brillouin/Raman microscope setup with eyes being measured in solution via a dipping objective. While laser light (red) is filtered out by Rubidium cells, Brillouin scattered light (purple dashed) of the sample and the methanol reference signal is analyzed by a 2-stage VIPA spectrometer and Raman scattered light (orange dashed) is analyzed with a commercial grating-based spectrometer. **(b)** Picture of the setup, where the eye is totally immersed in solution.

The combined Brillouin-Raman system is described in detail elsewhere (Rix et al., 2022). Briefly, it consists of a 780 nm wavelength excitation, coupled into an up-right WITec 300R microscope equipped with a ×10 objective (Zeiss Epiplan-Neofluar ×10/0.25NA) used for dehydration and hydration measurements and a ×40 water-dipping objective (Zeiss N-Achroplan ×40/0.75NA) used for CXL measurements (see Figure 1). The laser power on the sample was 25 mW. The Brillouin spectrometer consisted of a two-stage virtually imaged phased array setup (Berghaus et al., 2015). Axial scans consisting of 25 individual measuring points (with 1 s integration time and three repetitions per point) were performed in the center of the eye by elevating the stage up to 300 µm in z-direction. Note that the effective measuring depth is slightly greater (311 µm), because of the cornea’s refractive index being about 1.377 in the visible range (Scarcelli et al., 2012; Webb et al., 2020) resulting in an elevation of the image. However, this effect is small, because a water-dipping objective was used, which is manufactured for a refractive index of 1.33. Therefore, this mismatch is in the range of the axial resolution and thus neglected. Brillouin spectra were analyzed by custom-written (Rix et al., 2022) Matlab scripts (Matlab 2024a; MathWorks Inc., Natick, MA) based on Lorentzian function fitting in order to obtain the Brillouin frequency shift.

Additionally, confocal single point Raman measurements were performed in 50 µm depth, where the CXL effect is predominantly expected (Melcher et al., 2023; Ju and Tang, 2015). The acquisition parameters were set to 30 s integration time and 10 repetitions in order to obtain high quality (signal-to-noise ratio >28) Raman spectra, which is crucial for evaluating the subtle CXL effect. Raman spectra were subjected to baseline correction and normalized using Matlab’s *msbackadj* and *msnorm* functions.

### 2.3 Polarization-sensitive optical coherence tomography

**FIGURE 2:**
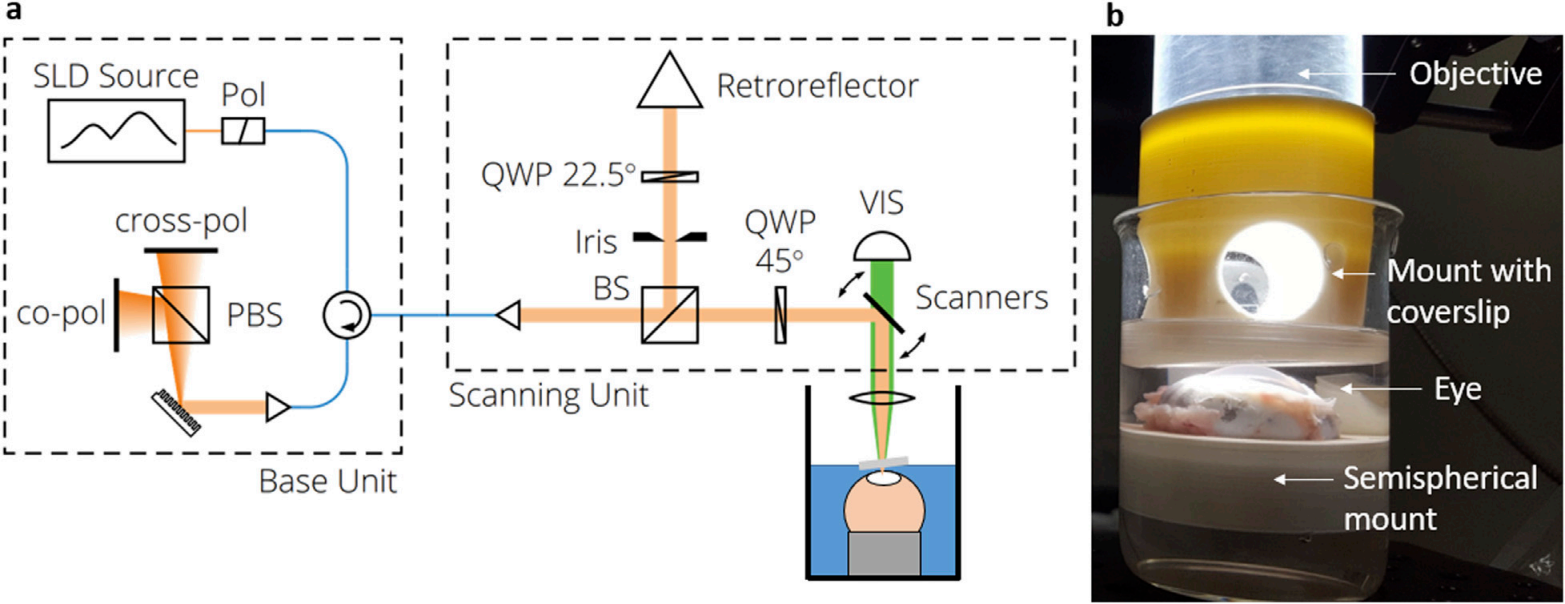
**(a)** PS-OCT setup consisting of the base unit, the beam scanning system and the scan lens kit. **(b)** Picture of the eye being placed in solution and measured through a cover slip added to the objective.

Thorlabs’ Telesto^®^ system (TEL220PSC2, Thorlabs GmbH, Germany) was used for PS-OCT measurements, which is described in detail elsewhere (Golde et al., 2021). It was operated at 28 kHz scanning rate and equipped with an LSM04 objective, on which a custom 3D-printed structure with integrated coverslip was mounted allowing for measurements in solution (see Figure 2). 3D volume scans were acquired in the center position of the corneas with a lateral scanning field of 2 mm in each direction and axial imaging depth of 3.5 mm (in air). The axial resolution was approximately 5 µm in stroma, assuming a refractive index of n = 1.376 for a wavelength of 1,300 nm according to (Tan et al., 2019). A custom-written segmentation algorithm was used to find the top and bottom edges of the cornea in order to calculate the central corneal thickness (CCT) considering the refractive index as well. Details on the segmentation algorithm applied to the OCT intensity images can be found in Supplementary Material. For the CXL measurements, the DOP weighted by taking the SNR into account was calculated from the PS-OCT data as described elsewhere (Baumann et al., 2018). For further analysis, a mean DOP was calculated by averaging over 50 subsequent, central A-scans in both lateral directions. Throughout this manuscript, it is referred to OCT when only the intensity information is considered, which might be collected also with a standard OCT device, while PS-OCT is mentioned in relation to polarization-specific interpretations. However, all evaluations are based on the same data collected with a PS-OCT device.

### 2.4 Statistical analysis

To account for the biological variance in the experimental data, the interquartile range (IQR) was calculated and illustrated as shaded area or error bar in the figures. Non-parametric Mann-Whitney U tests were performed with Origin (Origin 2024b; OriginLab Corp., Northampton, MA) to compare the groups. Differences were considered significant if p < 0.05.

## 3 Results

### 3.1 Dehydration measurements

**FIGURE 3:**
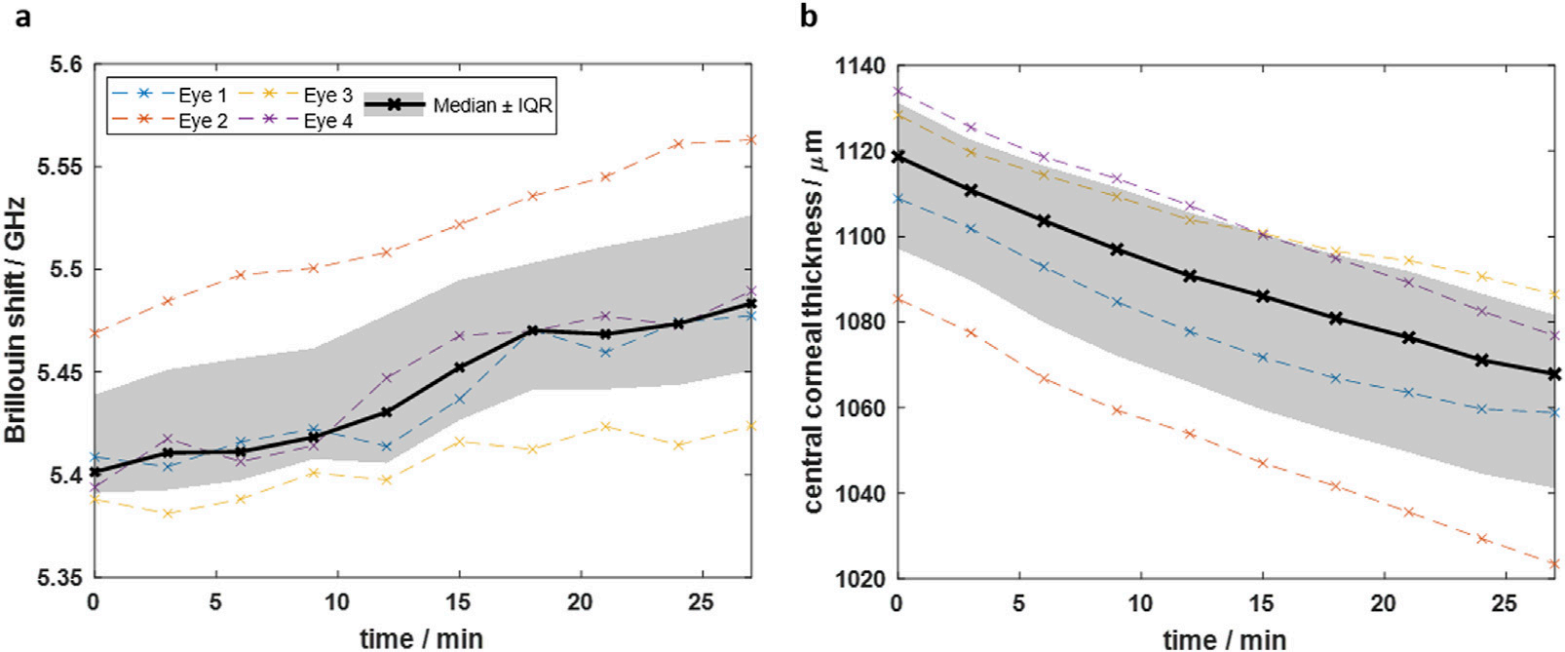
Time-dependence of dehydration process after de-epithelization showing that **(a)** the Brillouin shift increases and **(b)** the central corneal thickness decreases due to evaporation. Dashed colored curves are for individual eyes, whereas black solid curve is the median curve and the grey area the interquartile range, respectively.

For time-dependent dehydration investigations, BS and OCT measurements were performed every 3 min in a time range of 27 min on n = 4 eyes, respectively. On the one hand, axial BS scans were performed, whereof the Brillouin shift in 48 µm depth was extracted. This is in the same depth region where the single point RS measurements were performed later on in the CXL experiments. On the other hand, the CCT was evaluated using the OCT images. Both measures plotted as a function of the time after de-epithelization are shown in Figure 3.

Starting from a median value of 5.40 GHz (IQR: 5.39–5.44 GHz), the Brillouin shift increases over time predominantly linearly. The CCT follows an inverse behavior, i.e., starting from a median value of 1,119 µm (IQR: 1,097–1,131 µm) and decreasing almost linearly. The linear behavior indicates that after 27 min the dehydration process is still in full progress and the cornea is far away from an equilibrium state. It should be emphasized that already 5 minutes after de-epithelization, the Brillouin shift increases by 0.014 GHz while the CCT decreases by 12 μm, indicating the need for hydration stabilization for reliable measurements of biomechanical properties.

### 3.2 Equilibrium hydration

**FIGURE 4:**
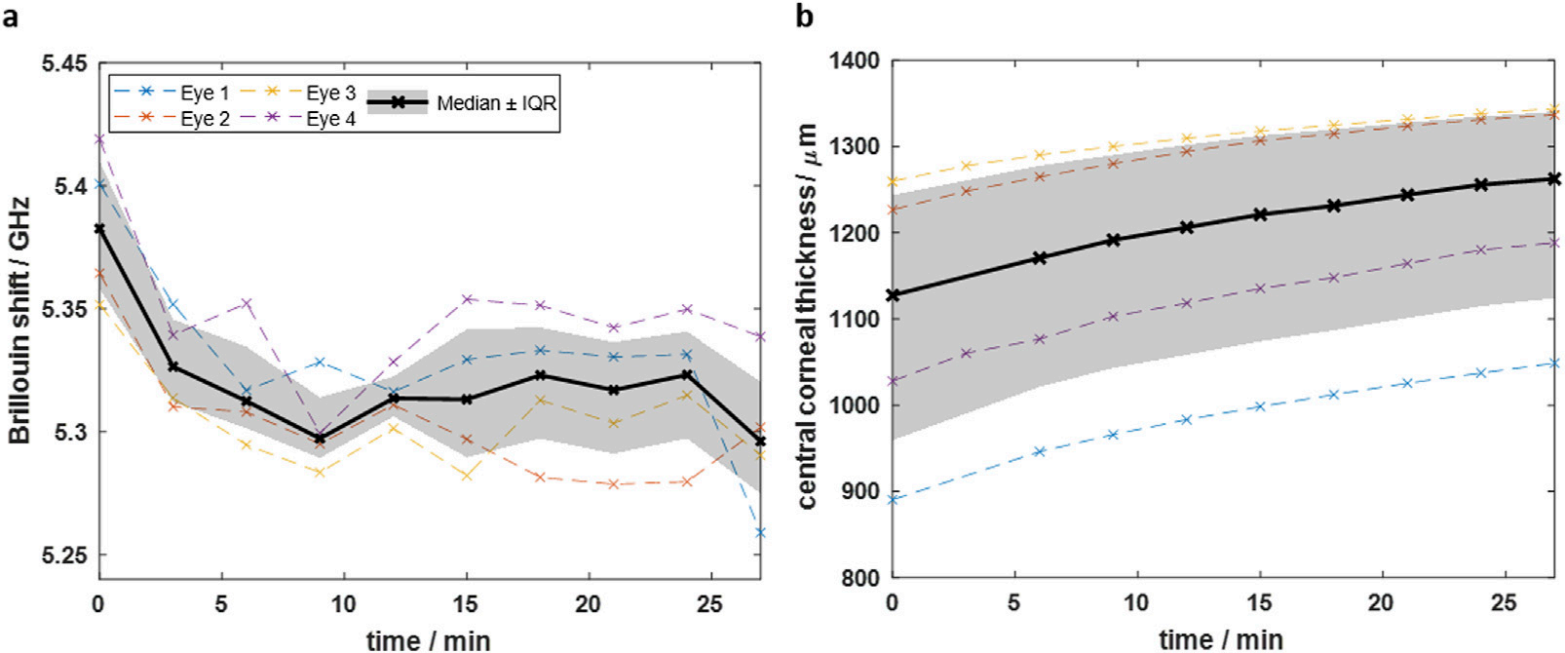
Time-dependent measurements after immersing eyes in BSS showing that **(a)** the Brillouin shift decreases and **(b)** the CCT increases over time. The Brillouin shift reaches an equilibrium, whereas the CCT still increases after 27 min. Dashed colored curves are for individual eyes, whereas black solid curve is the median curve and the grey area the interquartile range, respectively.

To achieve a balanced hydration state *ex vivo*, the eyes were placed in BSS. BS and OCT measurements were performed on n = 4 eyes each to assess the time required to reach a state of equilibrium. Figure 4a shows the Brillouin shift within 27 min after immersion of the eyes in BSS. The CCT within the same time is plotted in Figure 4b. The median data in Figure 4a shows that the Brillouin shift in 48 µm depth decreases exponentially shortly after immersion and reaches an equilibrium state at about 5.32 GHz (IQR: 5.29–5.34 GHz). Conversely, the CCT (Figure 4b) increases from 1,127 µm (IQR: 959–1,243 µm) to 1,262 µm (IQR: 1,124–1,340 µm) during 27 min soaking time, but converges to an equilibrium state, which is however not reached.

The results indicate that reliable Brillouin shift measurements with equilibrium hydration are possible, when immersing the eyes at least for 12 min in BSS. The fact, that the CCT is still increasing after 27 min might be due to the swelling of deeper tissue layers, where the water needs more time to reach. This is in accordance with the Brillouin shift in deeper layers (see Supplementary Material), which is still decreasing there and has not yet reached an equilibrium state after 12 min. However, as the CXL effect is mainly taking place in the upper part of the cornea, we chose hereinafter a soaking time of 15 min. This is sufficient to obtain a reliable Brillouin shift value for judging on the CXL effect, because an equilibrium state is already reached in the upper part of the cornea.

### 3.3 CXL measurements in BSS

**FIGURE 5:**
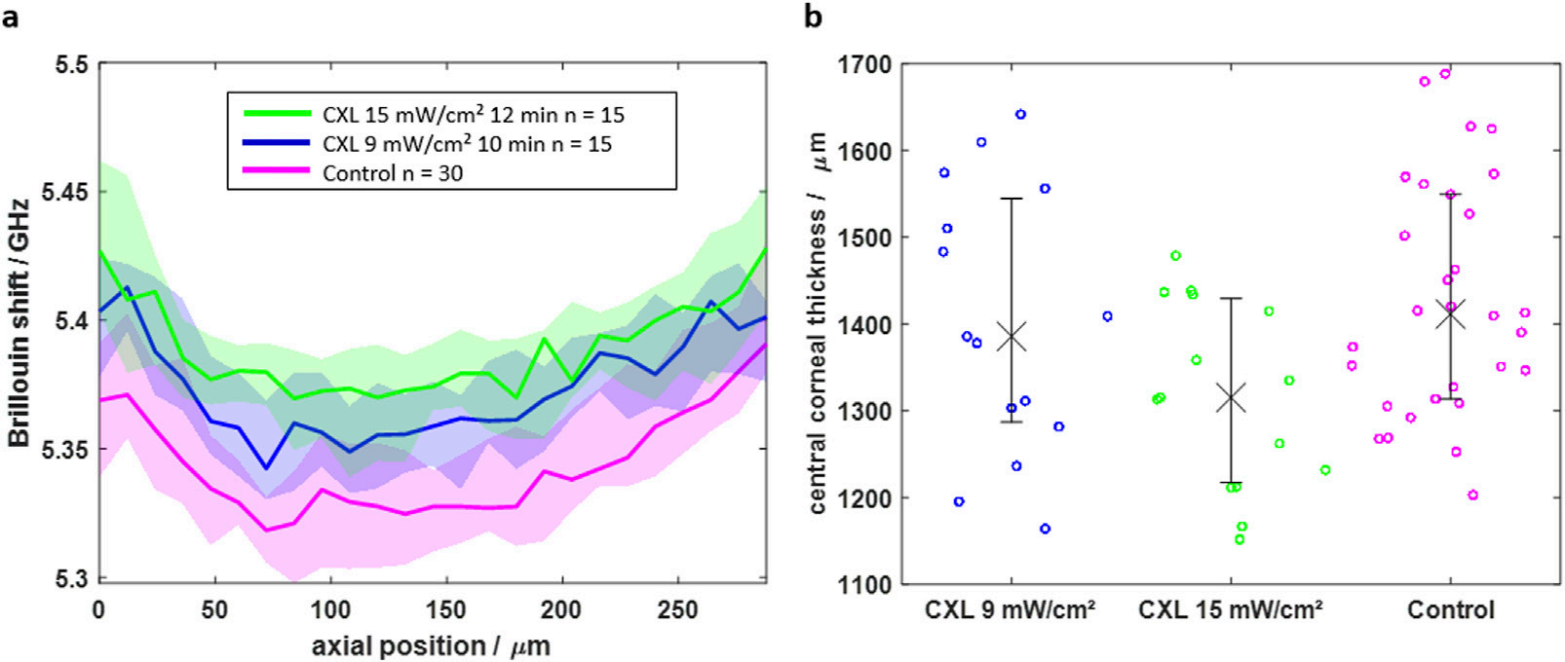
**(a)** Axial BS scans of CXL-treated (blue and green) and control (magenta) eyes indicating that CXL results in a higher Brillouin shift in the first 264 μm. **(b)** The central corneal thickness shows lower values for CXL-treated eyes, which is attributed to a mitigated water uptake. Solid line/crosses and shaded area/error bars are indicating the median value and interquartile range, respectively.

The finding that an equilibrium hydration state is reached at least in the upper corneal layer was transferred into practice for the measurements of the CXL effect. First BS/RS and afterwards PS-OCT measurements were conducted on the same eyes immersed in BSS. Fifteen paired eyes were treated with the 9 mW/cm^2^ protocol and another 15 paired eyes with the 15 mW/cm^2^ protocol, with one eye of each pair serving as control. All control samples were exposed to riboflavin, too. The two different protocols were used in order to evaluate whether a higher energy dose leads to an improved CXL effect. The Brillouin shifts of the axial scans are shown in Figure 5a.

For both CXL protocols, the Brillouin shift is significantly (Mann-Whitney U test, p < 0.05) higher for the CXL groups (blue and green) in the first 264 µm compared to the control group (magenta). In deeper tissue layers, the median Brillouin shifts of the groups approximate each other. Moreover, the 15 mW/cm^2^ protocol has a slightly stronger CXL effect, which gets mainly visible in a depth of 125 μm, where the Brillouin shift values are higher compared to those of the 9 mW/cm^2^ protocol.

The CXL shows an effect on the CCT (Figure 5b), too. It is lower for the CXL groups than for the control group (1,411 μm, IQR: 1,313–1,549 µm). Again, this effect is larger for the 15 mW/cm^2^ protocol (1,315 μm, IQR: 1,217–1,429 µm) than for the 9 mW/cm^2^ protocol (1,386 μm, IQR: 1,287–1,545 µm). Considering the hydration results from above, it is concluded that CXL alters the hydration conditions of the cornea influencing both, the Brillouin shift and the CCT.

**FIGURE 6:**
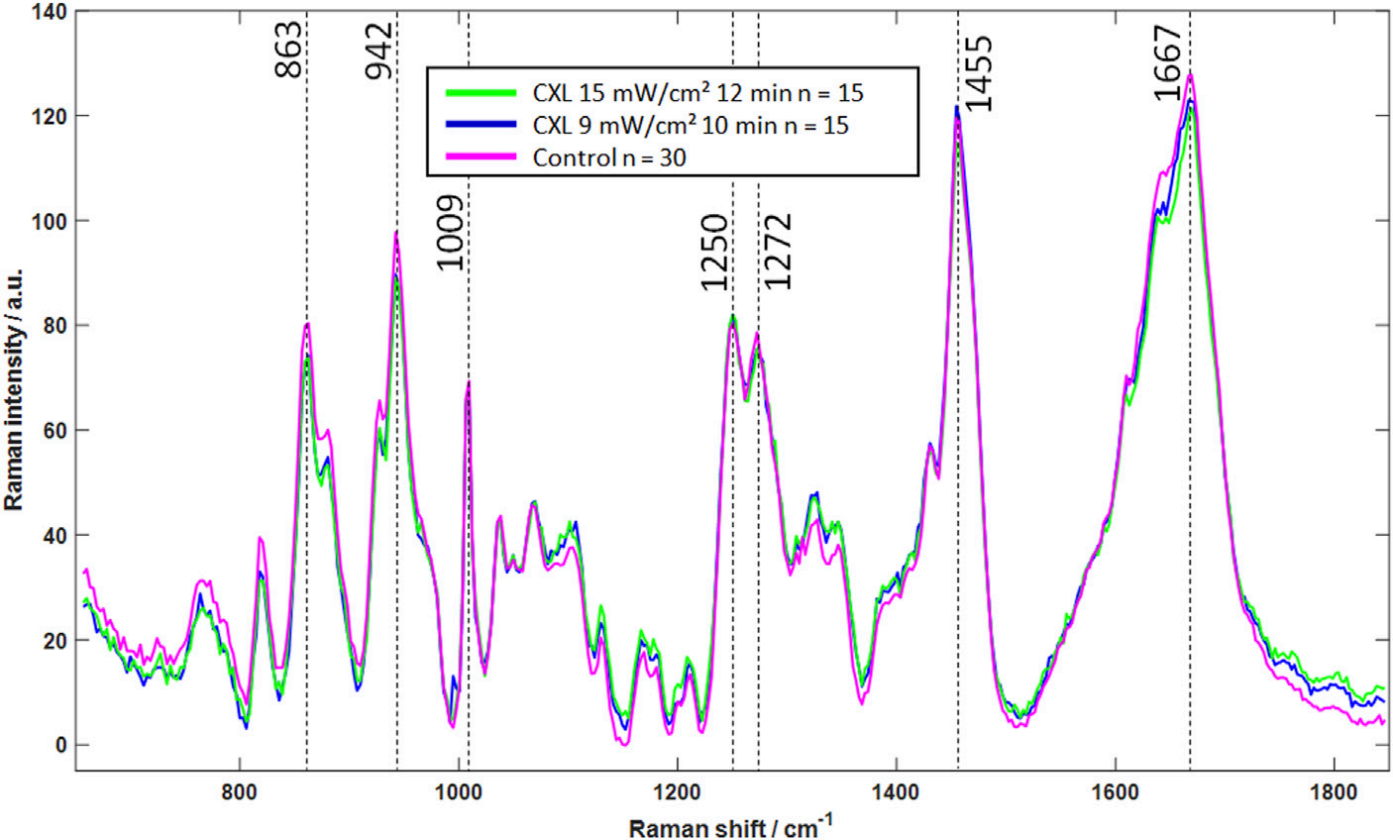
Mean Raman spectra of CXL-treated (blue and green) and control eyes (magenta), indicating that CXL does not lead to significant changes in Raman spectra. In particular, the strong signals (dashed lines) show no noticeable changes, which is an indicator of an unchanged molecular structure.

In contrast, the Raman spectrum shows changes in molecular structure, making the method a promising tool for evaluating the CXL effect independently of the hydration conditions. The mean Raman spectra of each group are shown in Figure 6. The overall spectral signature of the Raman mean spectra is similar within the three groups, which indicates that there is no change in the molecular structure of the cornea, i.e., the collagen backbone. The major bands at 863 cm^−1^, 942 cm^−1^, 1,009 cm^−1^, 1,250 cm^−1^, 1,272 cm^−1^, 1,455 cm^−1^ and 1,667 cm^−1^ show no clear changes. An assignment to functional groups is summarized in Table 1.

**TABLE 1:** Assignment of the Raman bands to functional groups.

Raman shift/cm^-1^	Assignment to	Reference
863	ν(C-C) hydroxyproline	Frank et al. (1995)
942	ν(C-C) of polysaccharides	Z. Yan et al. (2024)
1,009	Ring breathing of phenylalanine	Naumann, (1998)
1,250	Amide III	Frank et al. (1995)
1,272	ρ(CH_2_)	Schulz and Baranska, (2007)
1,455	δ(CH_2_)	Lau et al. (2003)
1,667	Amide I	Cheng et al. (2005)

**FIGURE 7:**
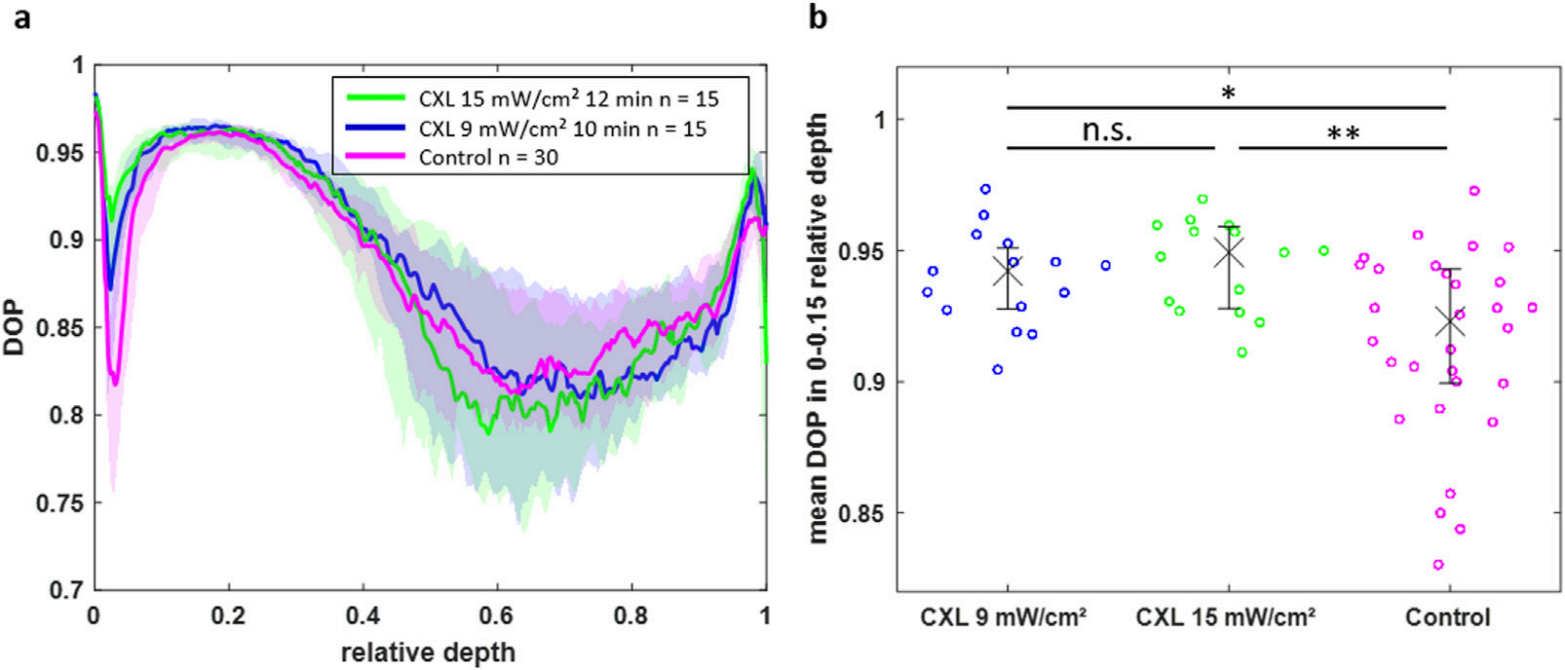
**(a)** DOP over the normalized depth showing that CXL-treated eyes have an increased DOP in the region of 0–0.15 relative depth. **(b)** Scatter plot of the mean DOP values in this region for all individual eyes indicating the statistical significance of the CXL effect on the DOP (*p < 0.05, **p < 0.01).

Although the BS reveals the CXL effect, the measurement times are much longer compared to OCT. Moreover, the additional feature of polarization-sensitive detection in PS-OCT allows for the evaluation of the CXL effect via the DOP. The DOP was determined on the same eyes and shown as a function of the depth in Figure 7a. To enable a comparison between corneas of different thicknesses, the DOP of each group is plotted against the relative depth. In the upper region between relative thicknesses of 0 and 0.15, the DOP is remarkably increased for the CXL-treated eyes ranging between 0.87 and 0.98, whereas the control eyes have DOP values down to 0.82. Thus, the CXL results in microstructural changes, so that the back-scattered light experiences a decreased depolarization compared to the control eyes. Again, the 15 mW/cm^2^ protocol (green) results in a stronger effect than the 9 mW/cm^2^ protocol (blue). In greater depths, the DOP is not distinguishable between the groups. In relative depths greater than 0.5 the high biological variability hampers a straightforward evaluation of the DOP.

For further statistical analysis, only the upper part of the corneas (relative depths between 0 and 0.15) was used, where the CXL effect is mainly occurring. For verification purposes, the mean DOP values of all eyes are visualized for the upper part (0–0.15 relative depth) in Figure 7b and for the lower part (0.15–1 relative depth) in Supplementary Material. The CXL-treated groups have a significant increased DOP in the upper cornea part compared to the control group (p = 0.0174 and p = 0.0022 for 9 mW/cm^2^ and 15 mW/cm^2^ protocol, respectively). However, the DOP is not significantly different between both CXL protocols. Moreover, the DOP is not significantly different within all groups in the lower part of the cornea.

### 3.4 CXL measurements in 16% dextran

All aforementioned measurements took place in BSS in order to guarantee an equilibrium hydration state. However, this does not reflect the *in vivo* conditions of human eyes during the CXL procedure, because there the eyes are in natural hydration state rather than in BSS. Therefore, further CXL measurements were performed in 16% Dextran solution, which fits the intrinsic hydration of porcine cornea (Fischinger et al., 2021). Again, first BS/RS and afterwards PS-OCT measurements were conducted on the same paired eyes. N = 12 eyes were treated with the 9 mW/cm^2^ protocol. The 15 mW/cm^2^ protocol was omitted here, as it showed only a slightly stronger CXL effect in previous measurements.

The axial Brillouin scans are shown in Supplementary Material. It is striking that the Brillouin shift values are about 0.35 GHz higher compared to the measurements in BSS (compare with Figure 5a), which agrees well with previous study of spheroids in Dextran (G. Yan et al., 2022). One factor that contributes to this increase is the refractive index n, which is directly proportional to the Brillouin shift and increasing by 1.72% when switching from pure BSS to pure 16% Dextran solution (Supplementary Material). However, this optical parameter is not explaining the full increase of the Brillouin shift (4.79%). Therefore, the mechanical properties of the cornea are assumed to be the main factor of the increase.

Moreover, there is no clear difference visible between the Brillouin shift of the CXL-treated (cyan) and the control (red) eyes. The two curves are overlapping especially when taking the interquartile range (shaded area) into account. Also, in the aforementioned region of 125 μm, where the CXL effect was maximal for BSS measurements, there is no striking effect visible for Dextran solution. There is only a slight tendency recognizable that the CXL-treated eyes have a tiny higher shift than the control eyes. The missing gap might be related to the mitigated/absent water uptake in the Dextran experiments compared to BSS experiments. The lower water uptake gets visible by evaluating the central corneal thicknesses.

In Dextran solution, the CCT (Supplementary Material) is only about 800 μm, which agrees well with the natural thickness of the cornea (HEICHEL et al., 2016). It is slightly less for the CXL group (783 μm, IQR: 752–830 µm) compared to the control group (810 μm, IQR: 786–844 µm).

The interpretation of the Raman spectra of measurements in dextran solution is somewhat complicated by the superposition of the dextran signals. Therefore, a factor analysis was first performed to separate the Raman signals from the dextran (further details are provided in the Supplementary Material). After subtraction of the Dextran contribution, the mean Raman spectra (Supplementary Material) show again that CXL treatment is not resulting in alterations of the molecular structure.

**FIGURE 8:**
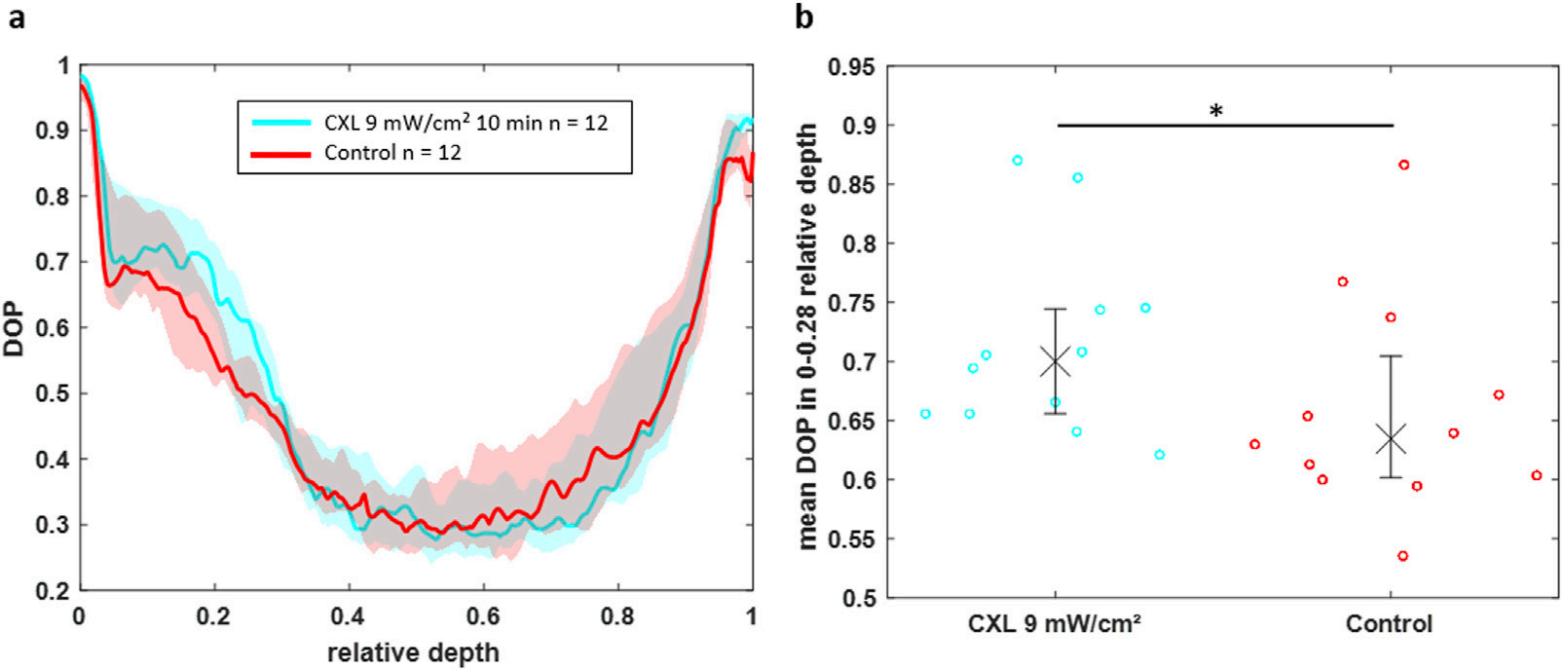
**(a)** DOP over the normalized depth showing that CXL-treated eyes (cyan) in 16% Dextran solution have an increased DOP in the region of 0–0.28 relative depth compared to control eyes (red). **(b)** Scatter plot of the mean DOP values in this region for individual eyes indicating the statistical significance of the CXL effect on the DOP (*p < 0.05).

The DOP value in the upper part of the cornea is again higher for CXL-treated eyes compared to control eyes (Figure 8a). Interestingly, in 16% Dextran solution the difference is observable up to a relative depth of 0.28, whereas it was only present up to a relative depth of 0.15 in BSS. This might be related to the absolute thickness, which is in BSS almost two times larger, whereas the CXL effect is limited to a fixed depth of approximately 250 µm. Statistical analysis revealed that the CXL effect on the DOP in the upper part (0–0.28 relative depth) of the cornea is again significant (Figure 8b, p = 0.0499).

## 4 Discussion

Brillouin microscopy as a novel full-optical technique has access to the biomechanics of tissue and is therefore a promising tool for *in vivo* evaluation of the CXL effect. However, its measure–the Brillouin frequency shift–is highly affected by the hydration state of the tissue (Wu et al., 2018; 2019; Jannasch et al., 2021). This impact was also observed in the present study. While evaporation after de-epithelization resulted in a dehydrated cornea, soaking in BSS rehydrated the cornea. In the first case, the Brillouin shift increased, whereas it decreased in the latter. This observation is explainable by the water retention of the cornea. The cornea constantly aims to be in a thermodynamic equilibrium with its environment. Therefore, water evaporates from the cornea into the surrounding air. De-epithelization even fosters this process (Çınar et al., 2014), whereas a moist-chamber attenuates it (Bao et al., 2018). On the other hand, positioning the eyes in water results in soaking (Meek, 2009). As a result, the cornea is either shrinking or swelling, when it does not fit the hydration conditions of the environment. This is in line with our findings from the OCT thickness measurements. Indeed, the CCT is decreasing due to dehydration and increasing due to rehydration.

For understanding the impact of the hydration state on the Brillouin shift, the morphological structure of the cornea has to be considered. It consists mainly of a collagen fiber network, where the fibers are predominantly aligned in-plane of the cornea and less perpendicular to it (out-of-plane) (Eltony et al., 2022; Abass et al., 2015). This leads to a layered structure, which is also visible in the OCT images (see Figure 1 in Supplementary Material). The multi-layered fiber network allows water to be trapped like in a sponge. Therefore, the cornea has to be treated as a composite consisting of collagen as a “solid” material and water as a surrounding fluid (Shao et al., 2018).

This composite model can be used to explain the observed behavior of the Brillouin frequency shift. During the dehydration and hydration experiments, only the fluid component changed, while the collagen component remained unchanged. As pure water and pure collagen have Brillouin shifts of 5.09 GHz (Berghaus et al., 2015) and 5.93 GHz (Palombo et al., 2014), respectively, dehydration results in a decreased water content and therefore in an increased Brillouin shift. In hydration experiments, the high water content leads to a decreased Brillouin shift.

On the contrary, the CXL procedure strengthens the “solid” collagen phase. As a secondary effect, this reduces the water retention potential. Therefore, less water can be trapped in the fiber network, wherefore the Brillouin shift is increased in the CXL-treated groups compared to the control group (Figure 5a).

By using 16% Dextran solution as soaking medium instead of BSS, the cornea is already close to or even at the thermodynamic equilibrium state and (almost) no additional water is stored in it. Therefore, corneas in 16% Dextran solution have a significantly lower thickness compared to soaking in BSS. In CXL experiments, the strengthened collagen fibers might again reduce the water retention potential. However, as the water uptake is only minimal, the Brillouin shift values between the CXL-treated and the control group are almost indistinguishable and the CCT is only slightly, but not significantly, decreased. Thus, BS gives mainly only indirect information on the CXL effect via the water storage potential rather than directly indicating a mechanical strengthening. This finding that the CXL effect is more difficult to detect with BS in medium-hydrated corneas compared to high-hydrated corneas is in line with previous study (Webb et al., 2020).

Whether in BSS or in 16% Dextran solution, registered Raman spectra revealed that CXL treatment is not altering the molecular structure of the cornea. Thus, new covalent bonds in form of cross-links between collagen fibers are not the reason for mechanical stiffening (Melcher et al., 2023). Therefore, it is instead assumed that the stiffening is achieved by polar-polar or hydrophobic-hydrophobic interactions between the collagen fibers. After CXL treatment, the amide I band shows a marginal broadening towards lower wavenumbers. This indicates new polar interactions of the amine group and supports the hypothesis that the stiffening of the cornea after CXL therapy is mainly due to new polar-polar interactions. Furthermore, it is assumed that these weak intermolecular forces are connected with the hydration of the cornea. This hypothesis is in line with the effects observed with BS, i.e., less water is stored in the fiber network after eyes are treated with riboflavin and UV light resulting in an increased Brillouin shift and an increased mechanical stiffness. However, the mechanism by which CXL treatment leads to a decrease in hydration remains unclear.

Moreover, PS-OCT was used in this study to verify, whether the CXL procedure was successful or not. As expected, the CXL-treated eyes show an increased DOP compared to the control eyes, being in accordance to previous literature (Ju and Tang, 2015). The DOP is mainly influenced by the collagen fiber structure and density (Ju and Tang, 2015). Two concurrent effects should be considered when interpreting these depolarizing properties of the cornea: On the one hand, collagen fibers are birefringent. Thus, randomly oriented fibers induce locally varying polarization states of the back-scattered light, which is observed as partially polarized or depolarized light. Accordingly, high DOP values are caused by highly oriented fibers, whereas lower DOP values are originating from more randomly oriented fibers. On the other hand, depolarization is also related to multiple scattering. Strong refractive index changes in the tissue cause this effect. While other tissues, e.g., demineralized tooth enamel with an increased pore volume, have shown similar relations between structure change and depolarization based on birefringence and multiple scattering (Grundmann et al., 2024), further investigations are required to better understand this interplay. However, these findings underline the hypothesis that after CXL treatment fibers are more densely aligned to build intermolecular forces resulting in a stiffening.

The advantage of BS and PS-OCT over classical stress-strain extensiometry is the depth information, which allows for an evaluation of the penetration depth of the CXL procedure. In our study, BS and PS-OCT revealed that CXL is limited to the upper part of the cornea, which agrees with previous literature (Ju and Tang, 2015; Kohlhaas et al., 2006). Both, BS and PS-OCT revealed a CXL penetration depth of about 250 µm.

The great benefit of all three optical methods over stress-strain extensiometry is the *in vivo* applicability. Although this study was performed *ex vivo*, important aspects for *in vivo* measurements can be derived. First, the corneal hydration has an effect on the Brillouin frequency shift. Although the cornea is normally almost constantly hydrated due to blinking (Shao et al., 2018), the eyes might dehydrate during long CXL-treating and measuring times. Second, Raman spectra revealed that not covalent bonds are the major reason for mechanical stiffening, wherefore weak intermolecular forces, which are highly affected by corneal hydration, are assumed to play a key role. This once again underlines the importance of a constant hydration state for any mechanical *in vivo* measurements. Third, PS-OCT is able to detect a significant CXL effect, is insensitive to corneal hydration and has a fast acquisition time. Moreover, OCT is already an established tool in eye clinics and PS-OCT requires only minor upgrades, thus making it a promising technique for future *in vivo* application.

## 5 Conclusion

Non-invasive Brillouin microscopy is a promising technique to assess the corneal biomechanics, for example, during the CXL procedure. However, it turned out that reliable measurements are only possible, when keeping the hydration state constant. Time series showed that evaporation in air immediately leads to increased Brillouin shift values, whereas soaking in balanced salt solution results in decreased Brillouin shift values coming along with corneal shrinking and swelling, respectively. After 12 min, the corneas are approaching a new equilibrium state, where reliable measurements are again possible.

CXL measurements of eyes immersed in BSS and 16% Dextran solution revealed that the effect of CXL on the Brillouin shift is more dominant in high-hydrated corneas compared to medium-hydrated corneas. This leads to the conclusion that BS is mainly only indirectly accessing the CXL effect by means of the water uptake, which is hampered in CXL-treated corneas. This suggests that weak intermolecular interactions are playing a main role in mechanical stiffening, which are difficult to detect with RS. In contrast, PS-OCT directly assesses the CXL effect via the fiber alignment of collagen and therefore being independent of the corneal hydration. Nevertheless, Brillouin microscopy remains a promising method for *in vivo* applications where hydration is kept constant by blinking.

## Data Availability

The datasets for this study can be found here: https://doi.org/10.25532/OPARA-750.
