# Supplementary material for: Investigation of corneal hydration and the impact of cross-linking therapy on water retention using Brillouin spectroscopy, Raman spectroscopy and polarization-sensitive optical coherence tomography
Source: Front Bioeng Biotechnol. 2025 Jun 24;13:1576809. doi: 10.3389/fbioe.2025.1576809 (PMC12235084; doi:10.3389/fbioe.2025.1576809)
Supplement: Supplementary file 1 [file DataSheet1.pdf]

Supplementary Material: Investigation of corneal hydration and the impact of cross-linking therapy on water retention using Brillouin spectroscopy, Raman spectroscopy and polarization-sensitive optical coherence tomography

Jan Rix[1, †,*], Svea Steuer[2, †], Jonas Golde[1,2,3], Fadi Husein[4], Felix Lochmann[4], Steven Melcher[2], Gerald Steiner[2], Roberta Galli[1], Julia Walther[1], Frederik Raiskup[4], Ramin Khoramnia[4], Robert Herber[4]

[1]TU Dresden, Faculty of Medicine Carl Gustav Carus, Medical Physics and Biomedical Engineering, Fetscherstrasse 74, 01307 Dresden, Germany

[2]TU Dresden, Faculty of Medicine Carl Gustav Carus, Anesthesiology and Intensive Care Medicine, Clinical Sensoring and Monitoring, Fetscherstrasse 74, 01307 Dresden, Germany

[3]Fraunhofer Institute for Material and Beam Technology IWS, Winterbergstrasse 28, 01277 Dresden, Germany

[4]Department of Ophthalmology, Faculty of Medicine and University Hospital Carl Gustav Carus, TU Dresden, Fetscherstrasse 74, 01307 Dresden, Germany

[†] These authors contributed equally to this work and share first authorship.

In this document, supplemental information to "Investigation of corneal hydration and the impact of cross-linking therapy on water retention using Brillouin spectroscopy, Raman spectroscopy and polarization-sensitive optical coherence tomography" is provided, which should help the reader to verify some minor important aspects of the study.

1 Segmentation algorithm for determining the central corneal thickness

A custom-written Matlab script was used for determining the central corneal thickness (CCT). For this purpose, first, a segmentation algorithm was implemented to identify the top (orange) and the bottom (yellow) of the cornea (cf. Figure S1a) in each cross-sectional OCT intensity image. The algorithm comprised the reduction of a central artifact in the middle third caused by a strong surface reflex leading to detector saturation, if present, removed horizontal artifacts by setting high mean intensities to zero, as well as the application of thresholds for the intensity and DOP. The Matlab function *imfill* was used to fill holes in the resulting connected area. Thereof, it was searched for the first pixel in axial direction in the image. The bottom was found by inverting the last half of the image and searching for the first pixel then. The segmentation was applied to all cross-sections of the entire volume stack (cf. Figure S1b). Top and bottom surfaces were then fitted with two-dimensional parabolas to create two hypersurfaces, from which the mean distance was calculated by averaging over the distances of a sub-volume from the center of 0.4 mm in both lateral dimensions (x and y in Figure S1b). The CCT was then calculated by dividing this distance by the refractive index.

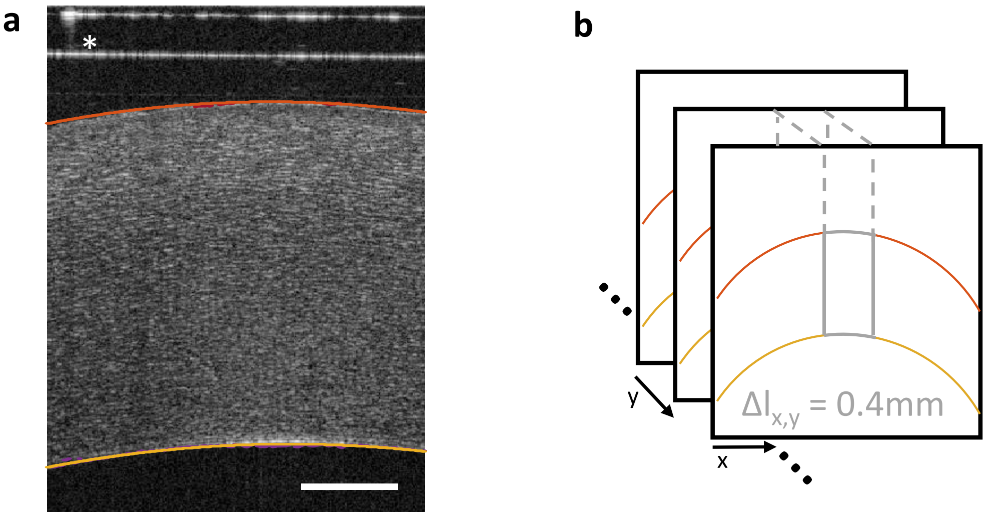

Supplementary Figure 1. a) Cross-sectional OCT intensity image showing the scattering distribution of the cornea under a thin glass plate (*). Orange and yellow lines indicate parabolic fits of the segmented top and bottom of the cornea. Scale bar corresponds to 0.5 mm in air. b) Segmentation was performed for all cross-sections within the scanned volume. The central corneal thickness was determined as averaged thickness of a sub-volume in the center marked in gray.

2 Brillouin shift in deeper layers

The time dependency of the Brillouin shift in 288 µm depth is depicted in Figure S2. It shows that even after 27 min after immersing in BSS, no equilibrium state is reach and the Brillouin shift is still decreasing. This is due to the water uptake of the cornea, which needs more time to reach the deeper tissue layers.

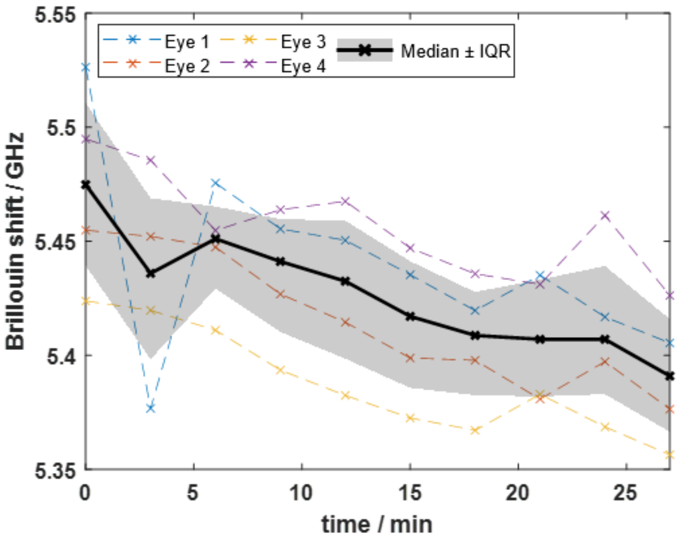

Supplementary Figure 2. Time dependency of the Brillouin shift in 288 µm depth showing a decrease even after 27 min.

3 Mean DOP of the lower part

For verification purposes, also the mean DOP of the lower part (0.15-1 relative depth) of the cornea was calculated. Here, statistical analysis shows no significant difference between the groups. This is attributed to the penetration depth of the CXL procedure being limited to the upper part (first approx. 250 µm).

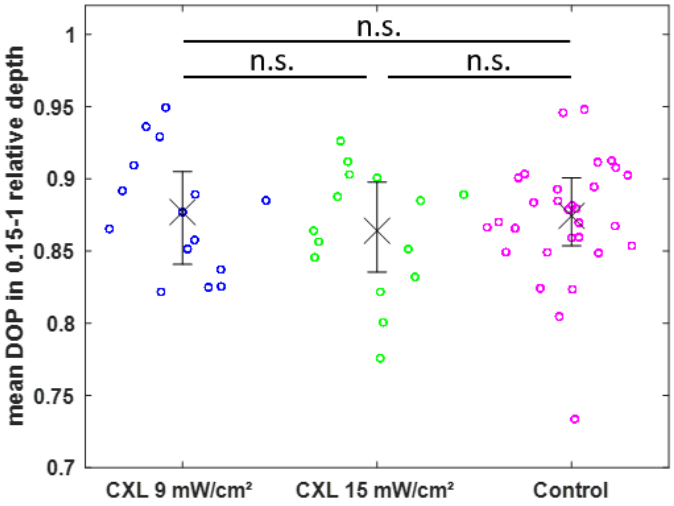

Supplementary Figure 3. Mean DOP of the lower part (0.15-1 relative depth) of the cornea showing no significant (n.s.) difference within the groups.

4 Brillouin shift and central corneal thickness of eyes in 16% Dextran

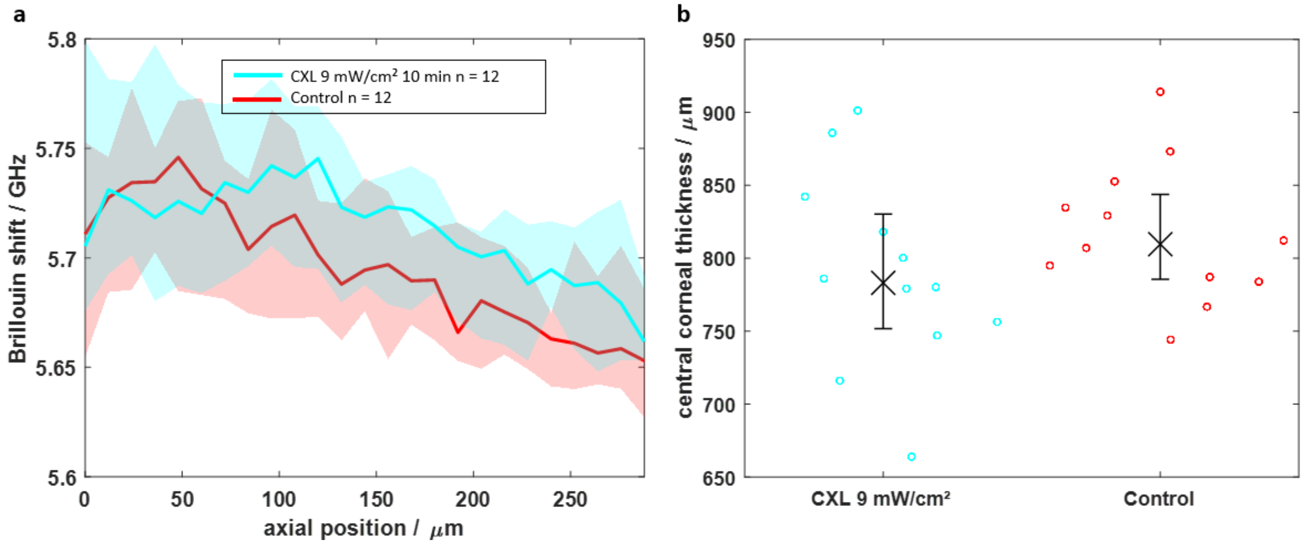

Supplementary Figure 4. a) Axial BS scans of CXL-treated (cyan) and control (red) eyes indicating that the previously observed Brillouin shift difference in the upper region is not present for measurements in 16% Dextran solution. b) The CCT of the CXL-treated eyes is only slightly lower compared to the control eyes.

5 Impact of the refractive index on the Brillouin shift

Since the refractive index is directly proportional to the Brillouin shift ($\nu_B = \frac{2\,n\,v_s}{\lambda}$), the latter is automatically increasing when switching from a solution with low refractive index to a solution with a high refractive index, even if the mechanical properties are not changing. In order to exclude that a refractive index increase is the only reason for the Brillouin shift increase of 0.35 GHz when switching from BSS to 16% Dextran solution as soaking medium, measurements of the Brillouin shift and the refractive index were performed of both solutions. The refractive index at 589 nm was determined by means of an optical refractometer (KERN ORL 94BS, Kern & Sohn GmbH, Balingen-Frommern, Germany). The values are plotted in Figure S5, where the error bars are indicating the technical precisions of 0.001 and 0.02 GHz, respectively.

Whereas the Brillouin shift shows an increase of 4.79%, the refractive index is only increasing by 1.72%. Therefore, it is assumed that the optical impact on the Brillouin shift via the refractive index is only minor. The major change is attributed to the mechanical properties.

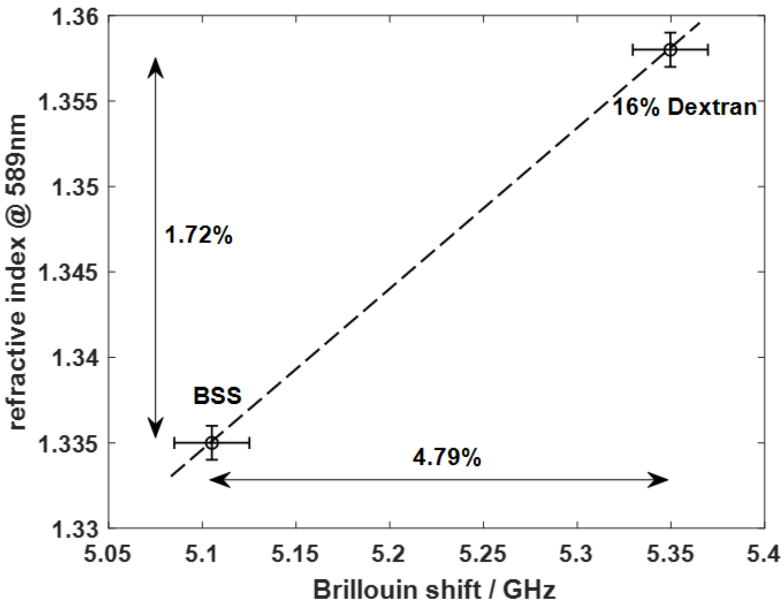

Supplementary Figure 5. Refractive index and Brillouin shift values for BSS and 16% Dextran solution indicating that the Brillouin shift increase (4.79%) cannot be explained by the refractive index increase (1.72%) alone.

6 Factor analysis of the Raman spectra in 16% Dextran solution

The Raman spectra of the corneas immersed in 16% Dextran solution consist of the vibrational bands of the cornea and those of Dextran. Since the bands are spectrally overlapping, an evaluation of the CXL effect is not straightforward possible, but a spectral decomposition is necessary. This was performed by fitting a pure Raman spectrum of Dextran (red curve in Figure S6) and a pure Raman spectrum of cornea tissue to the raw spectra, where the magnitudes of the pure spectra were set as optimization variables for a least-square fitting algorithm. Afterwards, the Dextran component was subtracted from the raw spectrum resulting in the corrected cornea spectrum (magenta).

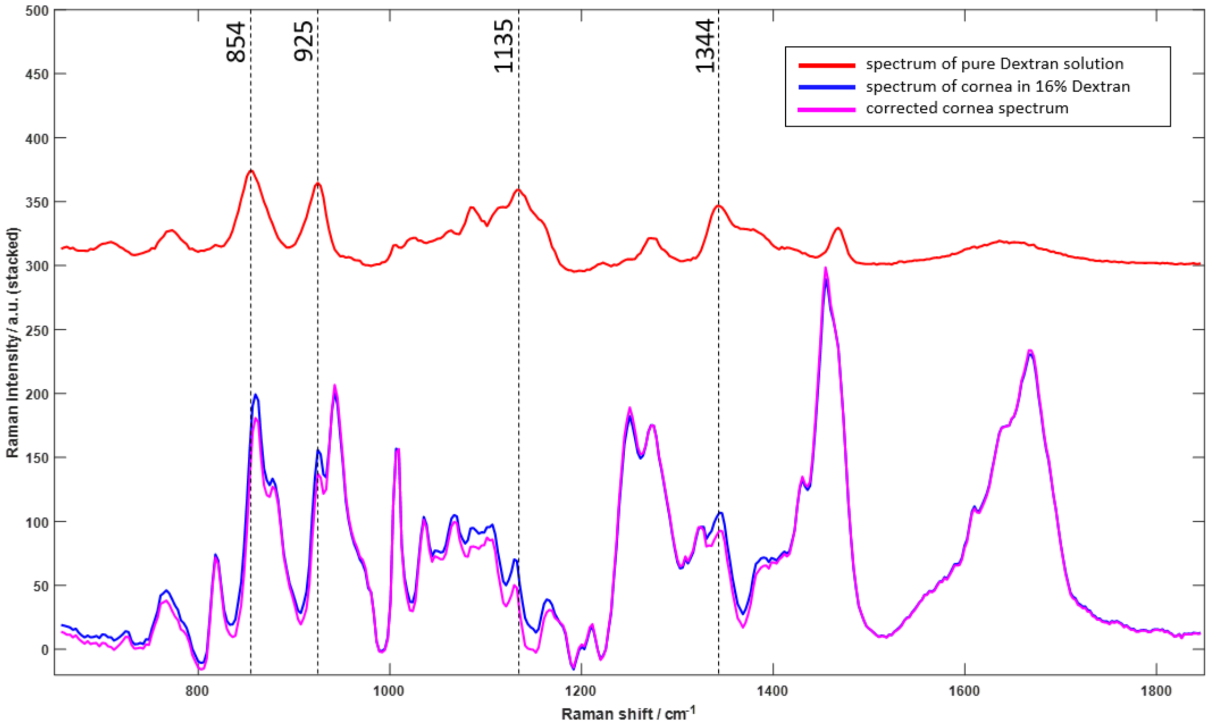

Supplementary Figure 6. Raman spectra of pure Dextran solution (red, stacked), cornea tissue in 16% Dextran (blue), and corrected cornea spectrum (magenta).

7 Raman mean spectra of eyes in 16% Dextran

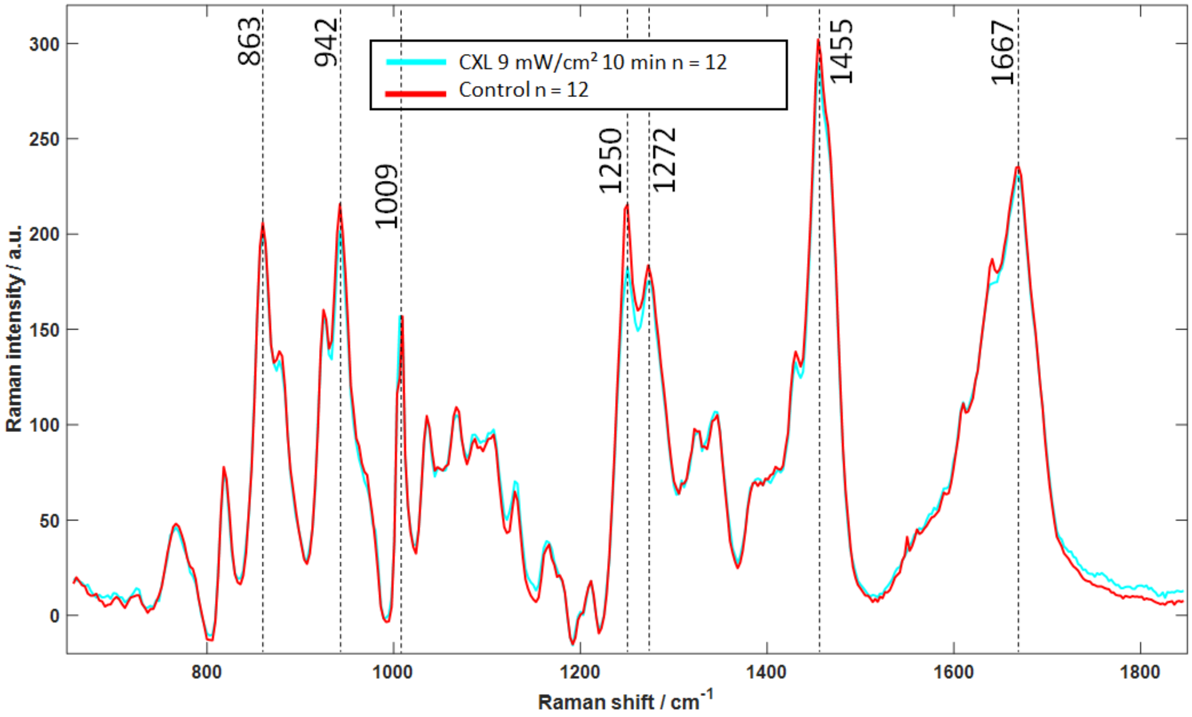

Supplementary Figure 7. Raman mean spectra of CXL-treated (cyan) and control (red) corneas measured in 16% Dextran solution show that the molecular structure of the cornea remains unchanged.
